# Dr. Henry

**Published:** 1836-10

**Authors:** 


### OBITUARY.

At Manchester, on the 5th September, Wm. Henry, m.d., one of the most distinguished of British chemists. Dr. Henry has long been most favorably known by his numerous writings, which are chiefly chemical. These extend over a period of nearly forty years. The following are some of his chief works, published separately; but he was a large contributor to the scientific journals of the day:—Experiments on Carburetted Hydrogen, *Lond.* 1797. 4to. On the Nature and Objects of Chemistry, *Manch.* 1799. 8vo. Epitome of Chemistry, *Lond.* 1801. 8vo. Elements of Experimental Chemistry, 2 vols. 8vo. *Lond.* 1810. On the Disinfecting Powers of increased Temperature, 1831, 8vo. An Estimate of the Philosophical Character of Dr. Priestley. *York*, 1832, 8vo. —Dr. Henry was as estimable in his private character as he was publicly honoured. His loss is extremely regretted in Manchester, of which place he was a conspicuous benefactor.

